# Autoantibodies in neuromuscular disorders: a review of their utility in clinical practice

**DOI:** 10.3389/fneur.2024.1495205

**Published:** 2024-11-01

**Authors:** Valentin Loser, Alex Vicino, Marie Théaudin

**Affiliations:** Department of Clinical Neurosciences, Nerve-Muscle Unit, Service of Neurology, Lausanne University Hospital and University of Lausanne, Lausanne, Switzerland

**Keywords:** autoantibodies, neuromuscular diseases, detection method, clinical practice, review

## Abstract

A great proportion of neuromuscular diseases are immune-mediated, included myasthenia gravis, Lambert-Eaton myasthenic syndrome, acute- and chronic-onset autoimmune neuropathies (anti-MAG neuropathy, multifocal motor neuropathy, Guillain-Barré syndromes, chronic inflammatory demyelinating polyradiculoneuropathy, CANDA and autoimmune nodopathies), autoimmune neuronopathies, peripheral nerve hyperexcitability syndromes and idiopathic inflammatory myopathies. The detection of autoantibodies against neuromuscular structures has many diagnostic and therapeutic implications and, over time, allowed a better understanding of the physiopathology of those disorders. In this paper, we will review the main autoantibodies described in neuromuscular diseases and focus on their use in clinical practice.

## Introduction

Autoantibodies are antibodies (ab) directed against self-antigens. They may be the direct cause of a pathological process (through activation of the complement cascade for example) or be detected as an epiphenomenon. Their main usefulness is to confirm a diagnosis suspicion, but some of them can also be used as severity or treatment response biomarkers. In neuromuscular disorders, several antibodies are highly sensitive and/or specific. In this review, we will describe the main autoantibodies used in the diagnostic process of neuromuscular diseases.

## Autoimmune neuromuscular junction disorder

### Myasthenia gravis

Myasthenia gravis (MG) is the prototype of post-synaptic neuromuscular junction disorder. Its pathophysiology involves immunological dysregulation, with autoimmunization beginning in the thymus. It is a humoral-mediated disease, associated with the presence of antibodies directed against some epitopes of the neuromuscular junction. The most frequently described antibodies are directed against the nicotinic acetylcholine receptor (AChR) and the muscle kinase (MuSK). The disease is characterized by fluctuating muscle weakness, typically affecting the ocular and bulbar regions, and sometimes involving the cervical and proximal limb muscles.

#### Anti-AChR antibodies

Serum anti-AChR-ab are of IgG1 and IgG3 subtypes and impair neuromuscular transmission by two main mechanisms: antigenic modulation by inducing endocytosis of the acetylcholine receptor and its lysosomal proteolysis, and its degradation by activation of the complement cascade and membrane attack complex (1, 2). The current reference method for detecting these antibodies is a radioimmunoassay (RIA) (3), but detection can also be performed by ELISA, with a lower sensitivity, reason why this method is less widely used routinely (4). These antibodies are detected in approximately 85% of patients with generalized MG and 50–60% in ocular MG, with a very good specificity (5). In the recently published multicenter SCREAM study, the specificity of anti-AChR-ab was 97.8% by RIA and 94.8% by ELISA (6). False positives have been described in patients with peripheral neuropathies, Guillain-Barré syndrome, ophthalmoplegia externa, neuromyelitis optica spectrum disorder and even in healthy controls. There appears to be no correlation between antibody titer and clinical severity, although ocular forms tend to have lower titers. There is no consensus on the use of anti-AChR-ab during follow-up; previous studies reported conflicting results about an association between changes in anti-AChR-ab serum levels and clinical severity (7–14). Monitoring anti-AChR-ab titers is therefore currently not indicated. Detection of serum anti-AChR-ab has two therapeutic implications. First, 10–15% anti AChR+ MG are associated with a thymoma, which, on the contrary, has not been described in anti-AChR- MG patients (15). Accordingly, thymectomy is recommended in patients with thymoma and anti-AChR+ MG (16). More recently, the MGTX trial demonstrated that thymectomy was also beneficial in a subgroup of patients under 65 years of age with non-thymomatous anti-AChR+ generalized MG (17). Those results confirmed the strong implication of the thymus, even in the absence of thymoma, in the pathophysiology of anti-AChR+ MG, and have been integrated into the latest international consensus guidance for management of MG (18). Secondly, severe refractory generalized anti-AChR+ MG may benefit from second-line therapy with complement inhibitors (eculizumab, ravulizumab, zilucoplan) (19–21) or antagonist of the neonatal Fc receptor (efgartigimod, rozanolixizumab) (22, 23).

#### “Low-affinity” anti-AChR antibodies

“Low-affinity” anti-AChR-ab can be found in patients with seronegative MG. Unlike anti-AChR and MuSK antibodies, which are high-affinity antibodies, low-affinity anti-AChR-ab are detected only by cell-based assay (CBA), by binding to a high-density AChR cluster (24–28). This detection method is less widely available and usually performed only in research centers. The detection rate of these antibodies in patients with double seronegative MG varies between studies but is probably close to 20% (26, 27). Specificity seems to be close to 100% (25). These antibodies are IgG1 and activate the complement pathway. Detection of low-affinity anti-AChR-ab seems to be associated with a less severe phenotype and a better response to immunotherapy (25). Low-affinity anti-AChR+ MG should probably be managed in the same way as anti-AChR+ MG, although clear recommendations are lacking.

#### Anti-MuSK antibodies

Serum anti-MuSK-ab are present in 30–60% of patients seronegative for anti-AChR-ab, representing 5–8% of patients with MG (29). These antibodies are predominantly IgG4, and induce functional blockade of the MuSK protein, without inducing antigenic modulation or activation of the complement pathway (30). The clinical phenotype of anti-MuSK+ MG may be different from anti-AChR+ forms, dominated by oculo-bulbar involvement, sometimes with amyotrophy and lingual fasciculations that may mimic amyotrophic lateral sclerosis with bulbar onset (31). The preferential involvement of certain muscles in anti-MuSK+ MG may reflect a different composition of the endplates in these muscles. These antibodies can be detected by RIA or ELISA, with a specificity close to 100% (6, 32, 33). CBA methods appear to be more sensitive but are not yet routinely available (34). Anti-MuSK-ab titer may correlate with disease severity and response to immunotherapy (35). It is exceptional for anti-MuSK-ab to be detected in the presence of anti-AChR-ab (36). Detection of serum anti-MuSK-ab has also significant therapeutic implications. In anti-MuSK+ MG patients, classical treatment options, including acetylcholinesterase inhibitors, corticosteroid-sparing immunosuppressive treatments, and intravenous immunoglobulins (IVIg), are usually less effective than in anti AChR+ MG (37). On the other hand, anti-CD20 therapy with Rituximab has shown efficacy in a few studies in anti-MuSK+ MG (38–41). Consequently, according to the latest international recommendations, Rituximab should be considered as an early therapeutic option in anti-MuSK+ MG patients, who have an unsatisfactory response to initial immunotherapy (18). As anti-MuSK+ MG are not related to thymus disorders, thymectomy is not recommended in this population (42). Moreover, complement inhibitors are probably not effective in anti-MuSK+ MG, as antibodies are predominantly IgG4, unable to activate the complement pathway.

#### Anti-LRP4 antibodies

LRP4 (lipoprotein-related protein receptor 4) is an endplate protein that, along with MuSK, is an agrin receptor and is required for AChR clustering and normal neuromuscular junction function. Antibodies to LRP4 have been detected in 7–33% of patients with double seronegative MG (43). Most of these are IgG1 antibodies, which can activate the complement pathway. Anti LRP4-ab are identified in 8% of patients with anti-AChR-ab, 15% of patients with anti-MuSK-ab, 4% of patients with other neurological autoimmune pathologies and in 0–4% of healthy controls (44, 45). Although initially considered to be relatively specific to MG, these antibodies were surprisingly detected in 10–23% of patients with amyotrophic lateral sclerosis (46). Indeed, LRP4 antigen appears to be also expressed in motor neurons and muscle, which may explain the presence of this antibody in motor neuron diseases. In some unclear situations, anti-LRP4-ab - cannot therefore distinguish reliably MG from amyotrophic lateral sclerosis. Anti-LRP4+ MG are usually associated with a less severe phenotype (ocular or mild generalized form) at disease onset, and a similar response to immunotherapy as anti-AChR+ MG patients (43). However, double positive patients (AChR/LRP4 and MuSK/LRP4) may have a more severe phenotype at onset (43). The favored detection method is a CBA.

#### Anti-striated muscle antibodies

Other antibodies are directed against intracellular muscle epitopes, known as anti-striated (striational) muscle antibodies. They recognize intracellular muscle proteins (titin, myosin, actin, ryanodine receptor or RyR); since these antibodies have no direct access to the antigen, they are unlikely to be pathogenic. Those antibodies are generally associated with anti-AChR-ab, making them of little diagnostic value. However, anti-titin and anti-RyR antibodies are present in most patients with thymoma (46). In particular, anti-titin was found in 80% of patients with MG and thymoma, especially in patients younger than 60-year-old (47). This good sensitivity in the presence of thymoma may therefore make these antibodies interesting biomarkers of thymoma, albeit with rather low specificity (39–70%) (48). Those antibodies are not specific for MG and may occur in patients with other autoimmune disease and in patients with thymoma without MG. Anti-titin and anti-RyR titers also appear to correlate with disease severity (49). The usefulness of anti-striated muscle antibodies testing in clinical practice is yet unclear.

#### Diagnostic workup

There are no clear international recommendations on the serological diagnostic approach to MG. Current consensus, underlined in the 2019 Italian recommendations, is to test for anti-AChR-ab and, if negative, for anti-MuSK-ab, in every clinical suspicion of MG (50). It is also recommended to repeat the assay at 6–12 months if initially negative (as antibodies can be undetectable at onset and become positive afterwards) (50). In Ho Chan et al., 15.2% of initially seronegative AChR MG patients became seropositive at 12 months (51). In double seronegative MG patients, clear recommendations are lacking. It is generally suggested to test for low affinity anti-AChR and/or anti-MuSK antibodies by CBA (if available), then for anti-LRP4 antibodies (50).

Testing for anti-MuSK antibodies need also to be considered in patients with bulbar involvement, suspect of ALS. According to Huijbers et al., anti-LRP4-ab testing in ALS patients may be considered in patients with prominent bulbar weakness, prolonged disease course, minor fluctuations and absence of upper motor neuron involvement (49).

### Lambert-Eaton myasthenic syndrome

Lambert-Eaton myasthenic syndrome (LEMS) is the prototype of pre-synaptic neuromuscular junction disorder. The disease results from the production of antibodies directed against voltage-gated calcium channels (VGCC), and is paraneoplastic in 50–60% of cases, mainly associated with small-cell lung cancer (SCLC) (52). Patient often present with a triad of lower extremity weakness, areflexia and autonomic dysfunction.

#### P/Q-type VGCC antibodies

P/Q-type (Ca_v_2.1) anti-VGCC-ab are present in 85–90% of patients with LEMS, and nearly 100% of paraneoplastic LEMS, associated with SCLC (53, 54). VGCC are expressed at the presynaptic cleft in the neuromuscular junction, and are also expressed by SCLC, suggesting that autoimmunization by the tumor is the cause of paraneoplastic LEMS (55). The subtype of VGCC antibodies is unknown, probably IgG1 and IgG3, capable of activating the complement pathway. The preferred detection method is RIA, with a probably good specificity. Although initially considered to be very specific to LEMS, it is not the case and P/Q-type VGCC-ab may be encountered in other neuroinflammatory diseases, such as paraneoplastic cerebellar degeneration (54), autoimmune encephalitis, myelopathies or inflammatory neuropathies (56). In one study, P/Q-type and N-type anti-VGCC antibodies were found in 1.7% of healthy controls and 4% of neurologically asymptomatic patients with SCLC (57). A high titer of P/Q-type anti-VGCC-ab (>1 nmoL/L) seems to be strongly correlated with an autoimmune neurological diagnosis, while low titers (0.03–0.09 nmoL/L) can be seen in other neurological disorders (56). The positivity of these antibodies must therefore be correlated with clinical and electrophysiological data. There seems to be no correlation between antibody titers and clinical severity.

#### N-type VGCC antibodies

N-type VGCC antibodies have also been reported in 33–49% of LEMS patients, usually in association with P/Q-type VGCC antibodies (54). Similarly to P/Q-type VGCC antibodies, those antibodies are more frequent in paraneoplastic LEMS. However, according to a recent study, the additional assay of N-type to P/Q type anti-VGCC may not significantly improve diagnostic performance (58). Indeed, among 93 patients with LEMS, 25 (26.9%) were positive for both anti-VGCC-ab type, 67 (72%) were positive for P/Q-type anti-VGCC-ab only and only one (1.1%) was positive for N-type anti-VGCC-ab only.

#### SOX1 antibodies

SOX protein are immunogenic antigens expressed in SCLC (59). They belong to the Sry-like high mobility group superfamily of developmental transcription factors and may be important for neurogenesis (60). Antibodies against SOX proteins have been identified in SCLC patients. Anti-SOX1-ab are found in 64–67% of patients with LEMS and SCLC (60–62). These antibodies are specifically associated with the presence of a SCLC and are absent in almost all non-paraneoplastic LEMS (specificity 95–100%) (60–62). In Sabater et al., none of the 50 idiopathic LEMS had anti-SOX1-ab (61). However, anti-SOX1-ab are not specific to LEMS, and may be found in other paraneoplastic neurological syndromes, or in SCLC alone; anti-SOX1-ab are therefore a marker of paraneoplastic neurological syndromes (61, 63). In a patient with LEMS, the detection of anti-SOX1-ab should therefore imply an aggressive search for SCLC. Anti-SOX1-ab are generally part of onconeural antibodies panels, and detection is made by immunoblot, confirmed by indirect immunofluorescence on tissue (tissue-based assay or TBA). However, TBA may have a lower sensitivity to detect anti-SOX1-ab compared to other onconeural antibodies, and confirmation with CBA of HEK cells expressing SOX1 is probably the gold standard (64). Of note however, anti-SOX1-ab CBA is only available in specialized centers.

Any clinical suspicion of LEMS should therefore be confirmed with a dedicated electrophysiological assessment and testing for P/Q-type anti-VGCC-ab. The usefulness of anti-SOX1-abtesting in a patient with confirmed LEMS has yet to be determined, but could imply a more aggressive search for cancer, particularly SCLC.

Characteristic of antibodies associated with neuromuscular junction disorders is summarized in Table 1.

**Table 1:** Characteristics of antibodies associated with neuromuscular junction disorders.

Disease	Antibody	Detection method	Sensitivity	Specificity	Indication	Correlation titer/severity	Titer follow-up
MG	AChR	RIA (ELISA)	85% of generalized MG 50–60% of ocular MG	RIA: 97.8% ELISA: 94.8% False+ in peripheral neuropathies, GBS, NMOSD, healthy controls	Every suspicion of MG. Repeat testing at 6–12 months if -	No	No
	MuSK	RIA ELISA CBA (if available)	30–60% of AChR- MG 5–8% of MG	ELISA & RIA: 99–100%	Every AChR- MG. Repeat testing at 6–12 months if - Consider in ALS patients with prominent bulbar weakness, prolonged disease course, minor fluctuations, and absence of upper motor neuron signs	Maybe	
	Low affinity AChR	CBA	20% of double seronegative MG	99–100%	Every double seronegative MG	Unknown	
	LRP4	CBA	7–33% of double seronegative MG	Probably high False+ in other neurological diseases, especially in 10–23% of patients with ALS	Every double seronegative MG		
LEMS	P/Q type VGCC	RIA	85–90% of LEMS 100% of LEMS with SCLC	Probably high (especially if >1.0 nmoL/L) False+ in other AE, SCLC and sometimes healthy controls	Every suspicion of LEMS	No	No
	SOX1	Immunoblot then CBA (if available) Immunoblot then TBA	64–67% of LEMS with SCLC 0–5% of LEMS without SCLC	Probably high False+ in other AE or in SCLC	Unclear	No	No

AChR, acetylcholine receptor; AE, autoimmune encephalitis; ALS, amyotrophic lateral sclerosis; CBA, cell-based assay; ELISA, enzyme-linked immunosorbent assay; GBS, Guillain-Barré syndrome; LEMS, Lambert-Eaton myasthenic syndrome; LRP4, lipoprotein-related protein receptor 4; MG, myasthenia gravis; MuSK, muscle kinase; NMOSD, neuromylitis optica spectrum disorder; RIA, radioimmunoassay; SCLC, small cell lung cancer; SOX1, Sry-like high mobility group box; TBA, tissue-based assay; VGCC, voltage-gated calcium channel.

## Autoimmune peripheral neuropathies

### Acute-onset autoimmune neuropathies: Guillain-Barré syndrome and variants

Guillain-Barré syndrome (GBS) is an acute-onset and monophasic inflammatory polyradiculoneuropathy, which includes different clinical variants et electrophysiologic patterns. The most frequent clinical phenotype is characterized by a proximo-distal tetraparesis, areflexia, and sensory involvement. It is characterized by an autoimmune attack on myelin, nodal/paranodal or axonal structures of the peripheral nerves and is usually post-infectious. The neural antigens targeted in this autoimmune process, particularly in “axonal” forms of GBS, are gangliosides, proteins expressed on axonal membranes, Schwann cells and myelin, where they participate in cell signaling and cell-to-cell communication (65). The main gangliosides are GM1, GD1a, Gd1b, GT1a and Gq1b. GM1 antigen is expressed mainly in the axolemma of nodes of Ranvier, the myelin of motor neurons and the dorsal ganglion (66), GD1a in the nodes of Ranvier of motor neurons (67), GT1a in the vagus and glossopharyngeal nerves (68), GQ1b in oculomotor nerves, neuromuscular spindles and possibly brainstem reticular matter (69) and GalNAc-GD1a in the axonal membrane of motor neurons and the axolemma of the sural nerve (70). Antibodies against gangliosides are detected in more than 50% of GBS patients (71). As each ganglioside has a different distribution in the peripheral nervous system, each antibody can be associated with a specific clinical phenotype.

#### Acute inflammatory demyelinating polyradiculoneuropathy

In acute inflammatory demyelinating polyradiculoneuropathy (AIDP), the classic form of GBS, there is no clear association with antiganglioside-ab, probably reflecting a rather cellular immune mechanism. It is therefore not recommended to test these antibodies in this subtype of GBS. Some studies have found anti-galactocerebroside (Gal-C), LM1 or GD1b antibodies in AIDP patients, but this association is unclear (72–74). A certain proportion of sera from AIDP patients binds to nodal and paranodal structure of the peripheral nerves, suggesting that yet unidentified antibodies may also play a role (75).

#### Acute motor axonal neuropathy

“Axonal” forms of GBS (in reality, nodopathies) classically includes acute axonal motor neuropathy (AMAN) and acute axonal motor and sensory neuropathy (AMSAN). AMAN is frequently associated with anti-GM1 and anti-GD1a IgG antibodies. In a recent Japanese-Italian collaborative study, 83% of patients with AMAN had anti-GM1, GD1a, GalNAc-GD1a or GM1b IgG (76). Pathogenesis of those antibodies, particularly anti-GM1 and anti-GD1a IgG, are the prototypic example of molecular mimicry (77). Almost half of patients with AMAN have a prior documented *C. jejuni* infection (78). *C. jejuni* contains in its lipo-oligosaccharides Gal(β1-3) GalNac, an epitope also present in GM1 and GD1a gangliosides; exposition to *C. jejuni* triggers an immune reaction with IgG1 and IgG3 production directed against gangliosides and causing the disease (79).

#### Acute ataxic neuropathies

Acute ataxic neuropathies include Miller-Fisher syndrome (MFS), Bickerstaff brainstem encephalitis (BBE) and acute sensory ataxic neuropathy (ASAN).

MFS is a variant of GBS characterized by ophtalmoparesis, ataxia and lower limb areflexia. Anti-GQ1b IgG antibodies were first described in 1992 in MFS patients (80). Few other studies confirmed the presence of anti-GQ1b in MFS, with a complete absence in normal and disease control groups, suggesting a high specificity (81). Anti-GQ1b-ab were later also associated with BBE (an overlap with MFS which includes a central nervous system involvement with disturbance of consciousness and corticospinal signs), acute ophthalmoparesis, or overlap syndromes between GBS and MFS/BBE (81). A comparative study found these antibodies in 83% patients with MFS and 68% patients with BBE (82). As for AMAN and AMSAN, cross-reactivity of anti-GQ1b IgG antibody with surface epitope on *C. jejuni* strains supports the hypothesis of molecular mimicry mechanism (83). Anti-GQ1b-ab cross-react with the structurally similar ganglioside GT1a.

ASAN is characterized by a sensory neuropathy with profound sensory ataxia and is often considered an incomplete form of MFS. It has been associated with anti-GD1b and, less frequently, anti-GQ1b IgG (84).

#### Pharyngeal-cervical-brachial variant

The pharyngeal-cervical-brachial (PCB) variant of GBS consists of an acute-onset bulbar paralysis and cervicobrachial weakness associated with upper limb areflexia. It is associated with anti-GT1a IgG in 50% of cases, some of these antibodies being able to cross-react with anti-GQ1b (85). Cross-reaction frequently occurs between GT1a and Gq1b-ab.

More recently, cases of severe GBS, with rapid tetraplegia, cranial nerve involvement, autonomic dysfunction and resistance to conventional treatments, have been associated with IgG1 antibodies to both neurofascin isoforms, known as anti-pan-neurofascin (86). Such antibodies, specific to the nodal regions, make these severe phenotypes of GBS enter the spectrum of autoimmune nodopathies, described in detail in the corresponding chapter.

### Chronic sensory-ataxic autoimmune neuropathies

#### Anti-MAG neuropathy

Anti-MAG neuropathy is a paraproteinemic neuropathy, characterized by a distal predominant demyelinating polyneuropathy mainly affecting large sensory nerve fibers, a phenotype previously known as DADS (distal acquired demyelinating symmetric neuropathy), and is associated with an IgM monoclonal gammopathy. MAG is a transmembrane glycoprotein localized in the myelin of Schwann cells and oligodendrocytes and plays an important role in myelin formation and axon-myelin interaction (87). Up to 50–65% of patients with a “DADS phenotype” neuropathy and IgM monoclonal gammopathy have IgM antibodies with anti-MAG activity (88). Historically, these antibodies were detected by western blot, but this technique has now been replaced by ELISA, with an improved sensitivity (89). The main issue with ELISA technique is a low specificity in case of low antibody titers. Indeed, using the manufacturer’s recommended cut-off of 1,000 Bühlmann titer unit (BTU), specificity is imperfect, with false positives in cases of chronic inflammatory demyelinating polyradiculoneuropathy (CIDP) for example (89). In a recent study, cut-offs of >1,000 BTU, >1,500 BUT and > 7,000 BTU were associated with a sensitivity of 100, 100 and 92.5% respectively, and a specificity of 90.99, 95.5 and 100%, respectively, to diagnose anti-MAG neuropathy (90). There is no clear correlation between antibody titer and disease severity. However, there seems to be a relationship between antibody titer and clinical response to treatment in responders; a > 50% titer reduction compared with its pre-treatment value might be a good indicator of therapeutic response (91). In patients with a clear electro-clinical diagnosis, anti-MAG titers can therefore be monitored, targeting a > 50% reduction in antibody levels (92).

A new ELISA technique, specifically targeting the HNK1 epitope of the MAG protein, has recently been developed, with a diagnostic sensitivity of 98% and specificity of 99% (93). With this technique, anti-HNK1 MAG antibody titers appear to correlate with the severity of neuropathy (93).

Anti-MAG IgM antibodies should be tested in any patients with a predominantly distal demyelinating neuropathy (DADS phenotype) and in the presence of IgM monoclonal gammopathy. According to EAN/PNS 2021 recommendations for CIDP, anti-MAG-ab should also be tested in all patients fulfilling CIDP diagnostic criteria and in presence of IgM monoclonal gammopathy (90, 94). The indication for anti-HNK1 MAG antibodies screening has yet to be clarified.

#### Chronic ataxic neuropathy with disialosyl antibodies

Chronic ataxic neuropathy with disialosyl antibodies (CANDA) is a rare syndrome characterized by a chronic sensory and ataxic, usually demyelinating neuropathy, occurring in the presence of an IgM monoclonal gammopathy reacting against gangliosides containing disialosyl epitopes (95). Some patients also present ophtalmoparesia, hence the term CANOMAD (chronic ataxic neuropathy with ophtalmoplegia, M-protein, cold agglutinins and disialosyl antibodies) (96). In CANDA/CANOMAD, IgM antibodies are directed against gangliosides harboring disialosyl groups, containing the sequence NeuNAc (α2-8)NeuNAc (α2-3)Gal, i.e., GD2, GD3, GD1b, GT1b, GT1a et GQ1b. The most frequently described antibodies are GD1b, GD3, GT1b and GQ1b, and most patients have antibodies reacting with multiple gangliosides (96–98). In the largest cohort of patients with CANDA/CANOMAD, 78% of cases had anti-GD1b autoantibodies, while other anti-disialosyl antibodies were each observed in less than 51% of patients (99). Those gangliosides are present in the neurons of the dorsal root ganglia and in oculomotor nerves, which explains the clinical symptoms.

### Chronic motor autoimmune neuropathies: multifocal motor neuropathy

Multifocal motor neuropathy (MMN) is an inflammatory neuropathy with a purely motor, slowly progressive and asymmetric involvement, generally affecting the distal limbs. One of its electrophysiological features is the presence of motor conduction blocks, distinguishing it from motor neuron disease, with which it shares many clinical features (100). The disease can be associated with the presence of specific IgM antibodies against the GM1 ganglioside (101). GM1 antigen is mainly expressed in the axolemma of the nodes of Ranvier, the myelin of motor neurons and, to a lesser extent, the dorsal root ganglion. It is unclear why, in MMN, the injury is restricted to motor nerves, even though GM1 is present is motor and sensory nerves. These antibodies appear to be pathogenic, firstly by inducing direct functional alteration of paranodal regions, and secondly indirectly by activating the complement cascade (102).

Anti-GM1 IgM are detected in around 50% (20–85%) of patients with MMN (102–104). High levels of antibodies seem to be relatively specific for MMN, while low levels however can be found in other inflammatory neuropathies, motor neuron disease or even in healthy controls (104). Detection of high titers of IgM anti-GM1-ab has very useful diagnostic implications, mainly in the differential diagnosis with other lower motor neuron syndromes, including amyotrophic lateral sclerosis, when motor conduction blocks are not present. However, gangliosides antibodies, including anti-GM1 IgM, have also rarely been described in amyotrophic lateral sclerosis, usually at low titers (105). There seems to be no correlations between antiganglioside levels and disease severity, nor necessity in monitoring antibody levels.

### Chronic inflammatory demyelinating polyradiculoneuropathy

CIDP is the chronic form of GBS, whose pathophysiology is less well understood, and may involve both humoral and cellular immunities. The presence of immunoglobulin and complement deposition in sural nerve biopsies from CIDP patients and the response to plasma exchanges and IVIg support the role of autoantibodies in CIDP pathogenesis (106). To date, no specific antibodies have been detected in patients with CIDP, apart from those directed against the nodal and paranodal regions of the nodes of Ranvier. However, patients carrying these antibodies are no longer classified as suffering from CIDP, but from autoimmune nodopathies (94).

### Chronic axonal polyradiculoneuropathies

Several neural antibodies can cause a chronic axonal polyradiculoneuropathy, including antibodies directed to collapsin response mediator protein-5 (CRMP5 [anti-CV2]), antineuronal nuclear antibody type 1 (ANNA-1 [anti-Hu]), amphiphysin, Purkinje cell cytoplasmic antibody type 2 (PCA-2 [microtubule-associated protein 1B (MAP1B)]) and adaptor-related protein complex 3, beta 2 subunit (AP3B2) (107). CRMP5 and amphiphysin IgG usually cause an asymmetric and painful polyradiculoneuropathy, usually axonal or mixed axonal-demyelinating, which may mimic CIDP (108, 109). A coexisting myelopathy (myeloneuropathy) has also been reported in some patients with CRMP-5 [anti-CV-2], amphiphysin, ANNA-1 [anti-Hu] and AP3B2 IgG (107). CRMP5 has been shown to be involved in axon-Schwann cell interaction, and is expressed in immature Schwann cells as well as in unmyelinated Schwann cells (Remak cells) (110). Little is known about the expression and localization of other neural antigens in the peripheral nervous system.

Testing for those antibodies can be considered in suspected CIDP patients with unexpected poor response to treatment, severe and rapid evolution, prominent axonal loss and association with other neurological signs (110). Most of these antibodies (ANNA-1 [anti-Hu], CRMP-5 [anti-CV-2], amphiphysin and PCA-2) are strongly associated with cancer (111). Testing those antibodies must be performed simultaneously in the serum and in the CSF to improve specificity (112).

### Antiganglioside antibodies testing: limitations and indications

Antigangliosides-ab are generally tested in a panel, containing the main gangliosides (GM1, GM2, GD1a, GD1b, GQ1b, GT1a, GT1b). The detection of antiganglioside-ab is difficult for several reasons: gangliosides are difficult to isolate based on their molecular weight or charge, making detection by western-blot complicated. Moreover, there is no reliable CBA for their expression. Finally, several gangliosides share the same backbone, leading to frequent cross-reactions. The gold standard technique for antigangliosides-ab detection is probably thin-layer chromatography, a technique not routinely available and requiring some expertise. Routinely detection is performed using ELISA commercial kits. Recommendations were published on how to best perform this ELISA (113). Immunodots or immunoblots are also used in certain settings, but their diagnostic performance is probably poorer (114).

According to the latest EAN/PNS 2023 guidelines, testing antigangliosides-ab is not recommended in most patient with typical sensory-motor GBS, because of their moderate diagnostic sensitivity and delay in result obtention (115). However, testing for those antibodies can be useful to confirm diagnostic suspicion in variant and atypical cases, and to rule out other disorders. For example, in pharyngeal-cervical-brachial variant, testing for anti-gangliosides-ab may be particularly useful in partial forms of the disease (e.g., acute bulbar palsy), in which a positive anti-GT1a may clarify the diagnosis. As nerve conduction studies cannot reliably distinguish between demyelinating and axonal form of GBS in the early phase of the disease, testing for antigangliosides-ab may help to make this distinction. Anti-GQ1b-ab testing is strongly recommended in MFS spectrum patients, because of their high sensitivity and specificity, and is integrated in the latest guidelines (115).

In the case of a chronic motor demyelinating neuropathy with conduction blocks on nerve conduction studies (suspicion of MMN), testing of anti-GM1 IgM antibodies is also required (116). Lastly, antigangliosides-ab screening is also indicated in CANOMAD/CANDA suspicion, i.e., in case of chronic ataxic sensory neuropathy with IgM monoclonal gammopathy, as the differential diagnosis of chronic sensory ataxic neuropathies are broad, including toxic, metabolic, mitochondrial or vitamin deficiencies (95). Testing for antigangliosides-ab is not recommended in CIDP (94).

Characteristics of antibodies associated with autoimmune neuropathies are summarized in Table 2.

**Table 2:** Characteristics of antibodies associated with autoimmune neuropathies.

Onset	Disease	Antibody	Detection method	Sensitivity	Specificity	Indication	Correlation titer/severity	Titer follow-up
Acute onset	AIDP	ø				Not recommended in classical sensory- motor GBS Recommended in variants (especially GQ1b in MFS spectrum) or atypical cases		
	AMAN/AMSAN	GM1/GD1a IgG Less frequently: GalNAc-GD1a and GM1b IgG	TLC ELISA	83% in AMAN	Good specificity if high titer (>50%) Poor specificity if low titer (ALS, immune neuropathies…)		No	No
	MFS spectrum	GQ1b IgG		83% in MFS 68% in BBE				
	ASAN	GD1b/GQ1b IgG		~50%				
	PCB variant	GT1a IgG		~50%				
Chronic onset	Anti-MAG neuropathy	MAG IgM	ELISA	>1,000 BTU: 99% >1,500 BTU: 99% > 7,000 BTU: 93%	>1,000 BTU: 91% >1,500 BTU: 96% >7,000 BTU: 100%	Every “DADS” neuropathy and/or CIDP with an IgM monoclonal gammopathy	No	Yes, target a > 50% drop in antibodies titer after treatment
		HNK1-MAG IgM	ELISA	98%	99%	Unclear	Maybe?	Unknown
	CANOMAD/CANDA	GD1b, GD3, GT1b and GQ1b IgM	TLC ELISA	GD1b: 78% Other: <51% Usually multiple gangliosides positivity	Good specificity if high titer (>50%) Poor specificity if low titer (ALS, immune neuropathies…)	Every chronic ataxic sensory neuropathy, especially if IgM monoclonal gammopathy	No	No
	MMN	GM1 IgM		20–85%		Every chronic motor neuropathy with conduction blocks	No	No
	CIDP	ø						
	Paraneoplastic	CRMP5, amphiphysin	Immunoblot confirmed by TBA	Unknown	Unknown	CIDP with poor response to treatment, severe and rapid evolution, axonal loss	Unknown	Unknown

AIDP, acute inflammatory demyelinating polyradiculoneuropathy; ALS, amyotrophic lateral sclerosis; AMAN, acute motor axonal neuropathy; AMSAN, acute motor and sensory axonal neuropathy; ANNA-1, antineuronal nuclear antibody type 1; AP3B2, adaptor-related protein complex 3, beta 2 subunit; ASAN, acute sensory ataxic neuropathy; BBE, Bickerstaff brainstem encephalitis; BTU, Buhlmann titer unit; CIDP, chronic inflammatory demyelinating polyradiculoneuropathy; CRMP5, collapsing response mediator protein-5; DADS, distal acquired demyelinating symmetric; ELISA, enzyme-linked immunosorbent assay; GBS, Guillain-Barré syndrome; HNK1, human natural killer-1; MAG, myelin associated glycoprotein; MFS, Miller-Fisher syndrome; MMN, multifocal motor neuropathy; PCA-2, Purkinje cell cytoplasmic antibody type 2; PCB, pharyngo-cervico-brachial; TBA, tissue-based assay; TLC, thin layer chromatography.

### Autoimmune nodopathies

The recent discovery of antibodies directed against nodes of Ranvier led to the definition of a new group of immune-mediated neuropathies: autoimmune nodopathies (117). They usually manifest as GBS or acute CIDP, usually in an aggressive form. Autoimmune nodopathies are thought to account for 5–10% of patients fulfilling diagnostic criteria for CIDP, and response to IVIg is usually poor. Of note, according to the latest EAN/PNS recommendations, patients harboring those antibodies are not considered having CIDP anymore (94).

In these auto-immune neuropathies, antibodies target nodal and paranodal cell adhesion molecules; contactin 1 (CNTN1) (118), contactin-associated protein 1 (Caspr1) (119), neurofascin 155 (NF155) (120), neurofascin 140 (NF140) and 186 (NF186), also called pan-neurofascin when all isoforms of neurofascin are targeted (panNF) (121). In most cases, nodal/paranodal antibodies are predominantly of the IgG4 subclass, known for its non-inflammatory properties. This can explain why response to IVIg is poor, and response to plasmapheresis and B-cell-depleting therapy is better. More rarely, antibodies of the IgG3 subclass have been reported in acute-onset auto-immunes nodopathies, such as panNF neuropathy. IgG3 have a strong proinflammatory effect and activate the complement pathway. In this case, treatment with IVIg can be beneficial. Nerve biopsy samples from patients with autoimmune nodopathy show paranodal detachment, without evidence of overt inflammation or demyelination, confirming that this immune neuropathy is pathologically very different from CIDP (122). Each antibody is associated with a specific clinical phenotype.

#### Neurofascin 155

NF155 antibodies are the most common among autoimmune nodopathies. Patients with IgG4 antibodies against NF155 share a similar phenotype: younger age of onset (mean age 42.4 years), rapid progression, distal motor involvement, ataxia with cerebellar features and a prominent low-frequency tremor (123). This antibody has also been associated with central nervous system demyelination, in addition to peripheral nerve pathology; “combined central and peripheral demyelination” (124). Response to IVIg or steroids is usually poor, whereas most patients respond to Rituximab. Anti-NF155 titers may correlate with clinical severity within the same patient, and anti-NF155 titers decrease in all Rituximab-treated patients (123).

#### Contactin 1

Patients with IgG4 anti-CNTN 1 also share a similar phenotype: an aggressive and acute neuropathy mimicking GBS, old age at onset, axonal features in nerve conduction studies, poor response to IVIg and nephrotic syndrome (125, 126). Anti-CNTN1 IgG3 antibodies have also been detected during the early phase and may explain why some patients respond initially to IVIg (127).

#### Caspr 1

Antibodies targeting Caspr1 or the Caspr1/contactin 1 complex have been less frequently reported. Patients usually presented with acute-CIDP, severe pain, axonal involvement in nerve conduction studies, a poor response to IVIg and a good response to Rituximab (128). The main antibody subtype is IgG4, but IgG4 antibodies were also detected during the early phase in a few patients with GBS.

#### Neurofascin 186

Nodopathy with anti-NF186 has also been rarely reported. Patients typically presented with a severe acute or subacute CIDP phenotype, characterized by predominant distal motor and sensory impairment, along with mostly demyelinating features in nerve conduction studies, without axonal loss (129). In the initial description, none of the patients exhibited tremors (which were commonly seen in NF155 nodopathy), and concomitant glomerulonephritis or retroperitoneal fibrosis was also noted (121). The main antibodies subtypes are IgG3 and IgG4, and some patients may respond to IVIg (130). Patient with specific NF186 antibodies being rare, it is debatable whether these patients should be characterized as a “pan neurofascin” nodopathy, with predominant reactivity to NF186 (130).

#### Pan neurofascin

Some patients with autoimmune nodopathies harbor antibodies interacting with all neurofascin isoforms, NF140, NF155 and NF 186; they are described as panNF neuropathy (86, 131). Antibodies may be of the IgG1, IgG3 or IgG4 subtypes, and patients usually displayed an aggressive and fast clinical progression. These patients exhibit a fulminant disease course, with a severe sensorimotor tetraplegia, severe cranial nerve involvement, autonomic dysfunction, often requiring mechanical ventilation due to severe respiratory insufficiency. Most patient have a monophasic course and are diagnosed as an “explosive GBS.” Response to IVIg is usually poor, whereas most patients respond to Rituximab (86).

#### LGI4

IgG4 antibodies targeting leucine-rich repeat LGI family member 4 (LGI4) have been recently reported in 4 Japanese patients with a subacute sensory-motor CIDP phenotype (132). Those antibodies need further validation.

#### Detection of nodal/paranodal antibodies

Detection of nodal/paranodal-ab is of clinical relevance, as it can identify a specific subgroup of patients who share some clinical characteristics and response to treatment very different from CIDP patients. Indeed, response to IVIg treatment is usually poor whereas most patients respond well to Rituximab. Antibodies to nodal/paranodal antigens are detected in the serum. The screening and confirmation assays used mainly depend on the laboratory, but it is highly recommended to confirm the positivity of an antibody with a second assay, to ensure its specificity (93). According to EAN/PNS latest recommendations, CBA with plasmids encoding for human recombinant proteins, ELISA and teased-nerve immunohistochemistry are the recommended methods (94, 114). CBA and ELISA are good screening techniques, and teased-nerve immunohistochemistry is usually performed as a confirmatory test. Detection of nodal/paranodal antibodies need to be performed in expert centers.

According to the latest EAN/PNS 2021 recommendations, testing for antibodies to nodal/paranodal antigens are advised in CIDP patients with the following features (94):

- Resistance to standard therapy with IVIg and corticosteroids.
- Acute or subacute aggressive onset, previous diagnosis of GBS or acute CIDP.
- Low-frequency tremor, ataxia disproportionate to the sensory involvement or other cerebellar features or predominantly distal weakness.
- Respiratory failure and cranial nerve involvement.
- Associated nephrotic syndrome.
- Very high CSF protein levels.

Characteristics of nodal/paranodal antibodies are summarized in Table 3.

**Table 3:** Characteristics of antibodies associated with autoimmune nodopathies.

Antibody	Detection method	Indication	Clinical characteristics
NF155	ELISA or CBA as screening method, TBA as confirmatory method	CIDP patients with the following features: Resistance to standard therapy with IVIg and corticosteroids Acute or subacute aggressive onset, previous diagnosis of GBS or acute CIDP Low-frequency tremor, ataxia disproportionate to the sensory involvement or other cerebellar features or predominantly distal weakness Respiratory failure and cranial nerve involvement. Associated nephrotic syndrome Very high CSF protein levels	Young age at onset (mean 42.4 years) Fast progression Distal motor involvement, ataxia with cerebellar features, low frequency tremor Combined central and peripheral demyelination Poor response to IVIg and steroids, response to Rituximab
CNTN1			Aggressive and acute progression, mimicking GBS Old age at onset NCS showing axonal features Poor response to IVIg Possible nephrotic syndrome
Caspr1			Acute-CIDP Severe pain NCS showing axonal feature Poor response to IVIg, response to Rituximab
NF186			Acute to subacute CIDP Distal motor and sensory involvement NCS showing mainly demyelinating features Some patients may respond to IVIg
PanNF			Aggressive and acute progression (“explosive GBS”) Sensorimotor tetraplegia, cranial nerve involvement, autonomic dysfunction, mechanical ventilation Poor response to IVIg, response to Rituximab

Capsr1, contactin-associated protein 1; CBA, cell-based assay; CSF, cerebrospinal fluid; CIDP, chronic inflammatory demyelinating polyradiculoneuropathy; CNTN1, contactin1; ELISA, enzyme-linked immunosorbent assay; GBS, Guillain-Barré syndrome; IVIg, intravenous immunoglobulins; NCS, nerve conduction studies; NF155, neurofascin 155; PanNF, pan neurofascin; TBA, tissue-based assay.

## Autoimmune neuronopathies

### Autoimmune autonomic ganglionopathy

Autoimmune autonomic ganglionopathy (AAG) is a severe immune mediated autonomic failure of acute-subacute onset, characterized by orthostatic hypotension, xerostomia, impaired pupil response, urinary retention, anhidrosis and gastrointestinal dysmotility. The disease is associated with the presence of ganglionic AChR type α3-ab, present in about 50% of patients (133, 134). Ganglionic AChR type α3 receptors are involved in synaptic transmission in autonomic ganglia. Those antibodies have pathogenic properties resulting in deterioration of synaptic transmission in sympathetic, parasympathetic, and enteric ganglia (134). High antibody titers >1.0 nmoL/L appear to be relatively specific for AAG (135), with a good correlation between antibody levels and the severity of autonomic involvement (133, 136, 137). Intermediate antibody levels are quite unspecific and may be found in cases of pure autonomic failure, postural orthostatic tachycardia syndrome (POTS) or gastrointestinal dysmotility (138). Low antibody levels (<0.2 nmoL/L) are non-specific and may be found in healthy controls (135). Three detection methods are available: RIA, Luciferase immunoprecipitation (LIP) and immunomodulation assay (139). RIA is the most widely established diagnostic method. A CBA method, which detects only potentially pathogenic antibodies, has recently been developed. In Karagiorgou et al., CBA was equally sensitive as the RIA, but was specific for AAG; all 15 patients with AAG and AChR type α3-ab were positive with CBA, and there was no false positive (140). This CBA assay should therefore replace RIA or LIP methods, but is still not yet widely available.

### Autoimmune and paraneoplastic sensory neuronopathies

Sensory neuronopathies are characterized by degeneration of the dorsal root sensory ganglion, and manifests as a pure sensory non-length-dependent neuropathy. Acquired forms are due to a variety of dysimmune, toxic, paraneoplastic or idiopathic etiologies (141). Sjögren syndrome is the most frequent dysimmune cause of sensory neuronopathy, and sensory neuronopathy is the most frequent and typical neuropathy of Sjögren syndrome (142).

#### Anti-FGFR3 antibodies

In 2015, antibodies directed against the intracellular domain of fibroblast growth factor receptor 3 (FGFR3) have been described (143). In a multicenter prospective study, 15% of patients with a sensory neuropathy had FGFR3 antibodies; 2/3 of patients fulfilled the criteria for sensory neuronopathy, 17% for small-fiber neuropathy and 19% for other sensory neuropathies, the majority (89%) being non-length-dependent phenotypes (144). Patients with anti-FGFR3 sensory neuronopathy have usually a progressive disease-onset, symmetric involvement of 4 limbs, impairment of all sensory modalities with neuropathic pain, and a motor involvement in 25% of cases (144). IVIg are effective in 80% of cases (145). In adult rats, FGFR3 was expressed in small and large sensory neurons of the dorsal root ganglia, and activation of the receptor could play a role in regulation of many cell functions including survival (146). It is yet unknown whether those antibodies play a pathogenic role or are only biomarkers of a dysimmune process. However, FGFR3 antibodies recognize the intracellular domain of the protein, and should these antibodies be able to enter the neurons (which is yet unknown), they could interfere with signal transduction and dorsal root ganglia function. ELISA is used to detect those antibodies.

#### Anti-AGO1 antibodies

In 2021, antibodies to argonaute proteins 1 and 2 (AGO1 and 2) have been identified in immune diseases of the central and peripheral nervous systems, in particular sensory neuronopathies and limbic encephalitis (147). These antibodies are not specific to neuronopathies. The presence of these antibodies was strongly associated with an autoimmune context, particularly Sjögren’s syndrome. In a recent retrospective study, 13% of patients with sensory neuronopathy had anti-AGO1 antibody, which was associated with a more severe phenotype, but a better response to immunotherapy with IVIg (148). AGO are involved in degradation of RNA. The site of tissue expression of AGO proteins is not well known; it is unclear whether AGO antibodies have a pathogenic role directly in the dorsal root ganglia, or whether they are only a biomarker of dysimmune process (147, 148). AGO antibodies are detected by ELISA.

#### Paraneoplastic forms

Sensory neuronopathies are among the most common paraneoplastic neurological syndromes and have been associated with ANNA-1 [anti-Hu], CRMP-5 [anti-CV2], PCA-2 [MAP1B] and amphiphysin (108). Some paraneoplastic sensory neuronopathies are seronegative, especially those associated with hematologic malignancies (149). The most frequent paraneoplastic form of sensory neuronopathy is Denny-Brown syndrome, which is associated with ANNA-1 [anti-Hu] antibodies (150, 151). Anti-Hu paraneoplastic syndromes can manifest with various clinical syndromes. In a series of 27 patients with anti-Hu syndromes, 20 (74%) had a clinical neuropathy (most frequently a pure sensory neuropathy) (152). Anti-Hu recognizes a family of RNA-binding proteins expressed in the nuclei of neurons and SCLC cells; in neurons, they have a role in the development and maintenance of the neuronal phenotype (153). There is no evidence that the anti-Hu antibodies are pathogenic, they may rather be a marker of dysimmune reaction. Anti-Hu antibodies are usually identified in patients’ sera but can also be identified in the CSF. In patients with peripheral neuropathy, CSF anti-Hu titers are probably lower compared to patients with encephalomyelitis (154). The identification of anti-Hu antibodies strongly predicts an underlying cancer, most commonly a SCLC, with an estimated sensitivity of 82% and specificity of 99% (155). There is probably no correlation between Hu antibodies titers and neurological outcome; there is therefore no indication for monitoring antibody levels (156).

### Autoimmune and paraneoplastic motor neuronopathies

Motor neuronopathies (or motor neuron diseases) manifest as a pure motor, symmetric or asymmetric, neuropathy. They are rarely of autoimmune origin and are frequently neurodegenerative (as in ALS) or, more rarely, of an infectious origin (as in poliomyelitis).

#### IgLON5

Ig-like cell adhesion molecule 5 (IgLON5) antibodies have been first described in 2014 in patients presenting with sleep apnea, REM-sleep or non-REM-sleep disorders (157). In 2021, 5 patients with a bulbar onset motor neuronopathy were found having serum IgLON5 antibodies, 4/5 patients having a coexisting sleep disorder (158). In another series, all patients had a coexisting neurologic finding, especially a vocal cord paresis, chorea or REM sleep behavior disorder (159). Most patients have IgLON5 antibodies in the serum, and a few also in the CSF. Anti-IgLON5 antibody testing is performed using a CBA (157). An early immunotherapy, prior to advanced neurodegeneration, seems to be efficient and associated with a better long-term clinical outcome (160). No paraneoplastic forms have yet been described.

#### Other antibodies

Paraneoplastic motor neuronopathies have also been rarely described in association with anti-Ma2 and ANNA-2 [anti-Ri] antibodies (161, 162). More recently, leucine zipper protein 4 (LUZP4) antibodies have been described in patients with neurological paraneoplastic syndromes, presenting as rhombencephalitis, limbic encephalitis, seizures and/or motor neuronopathy (163). Ma2 and ANNA-2 antibodies are included in screening panels for onconeuronal antibodies, detected usually by immunoblot, confirmed by TBA. LUZP4 antibodies testing is not routinely available.

## Autoimmune small fiber neuropathies

Small fiber neuropathy (SFN) is characterized by isolated involvement of A *δ* and C small nerve fibers, causing neuropathic pain, reduced sensation to pain and temperature, and dysautonomia. Similarly to sensory neuropathies and neuronopathies, SFN can be caused by a variety of metabolic, toxic, infectious, inherited and dysimmune etiologies, and cryptogenic forms are frequent (164). Sjögren syndrome, less frequently systemic lupus erythematosus and sarcoidosis, may be responsible for autoimmune SFN (164). Paraneoplastic neurological syndromes with ANNA-1 [anti-Hu] and CRMP5 [anti-CV2] antibodies can also cause a small fiber involvement, but rarely isolated (109, 110). According to de Greef *et al*, up to 19% of patients with cryptogenic SFN may have underlying immunological abnormalities, suggesting an immune component (165). Diagnosing autoimmune SFN is important since immunotherapy may be beneficial in those patients.

### Anti-FGFR3 and anti-TS-HDS antibodies

As mentioned previously, anti-FGFR3-ab have been described in patients with sensory neuronopathies, 17% of them fulfilling a SFN diagnosis (144). In a recent retrospective study, 17% of patients with SFN and dysautonomia had anti-FGFR3 antibodies (166). Those antibodies are not specific to SFN and are usually rather found in neuronopathies.

Trisulfated heparan disaccharide (TS-HDS) is a disaccharide component of the glycosylation moieties of heparan and heparan sulfate and is expressed in peripheral nerves surface. IgM antibodies binding to TS-HDS have been first described in patients with a painful, predominantly sensory neuropathy (167). In a recent retrospective cohort of cryptogenic SFN, 37% of patients had IgM anti-TS-HDS-ab, and 15% had IgG anti-FGFR3; the presence of anti-TS-HDS defined a subset of female patients with acute-onset, and non-length dependent SFN (168). In another study, 28% of patients with SFN and dysautonomia had anti-TS-HDS-ab; most of these patients presented with dysautonomia, but 30% of them had a normal intraepidermal nerve density in skin biopsy (166). The specificity of anti-TS-HDS antibodies for autoimmune SFN is still questioned. In a recent real-world study, among 77 patients with TS-HDS antibodies, 34% did not have evidence of neuropathy and 12% had another known cause of neuropathy (169).

Data about response to immunotherapy in TS-HDS/FGFR3+ SFN are conflicting. In two studies, most patients with those antibodies improved with immunotherapy, either IVIg or plasma exchange, with a significant reduction in pain scores (170, 171). In Chompoopong P et al. however, response to immunotherapy was observed only in 31% of TS-HDS positive patients and was not higher than in TS-HDS negative patients (169). More recently, a double-blind placebo-controlled pilot study did not show any benefit of IVIg treatment in SFN patients with anti-TS-HDS and/or FGFR-3 antibodies (172). In conclusion, anti-FGFR3/TS-HDS antibodies may not be specific to SFN, and their pathogenicity in neuropathies has yet to be proven in animal or cell culture models. Nevertheless, detection of such antibodies can give a clue about a potential autoimmune etiology/context and may prompt immunotherapy, even though response to immunotherapy is uncertain. Those antibodies can be detected with ELISA (144, 169).

### Anti-plexin D1 antibodies

Anti-plexin D1 IgG are antibodies binding selectively to mouse unmyelinated C-fiber neuron in the dorsal root ganglia. Those antibodies have been initially described in a fraction of patients with neuropathic pain and neuroinflammatory diseases, and in patients with idiopathic trigeminal neuropathy (173, 174). Recently, plexin-D1 IgG were found in 12.7% (8/63) of patients with probable SFN and in 0% of healthy controls (175). Those antibodies seem to be pathogenic, according to their mechanism of action, and may be more specific to SFN than FGFR3 and TS-HDS antibodies. Response to immunotherapy of anti-plexin D1+ patients is currently unknown. Those antibodies can be detected with ELISA (175).

### Other antibodies

Other novel antibodies have been recently described in idiopathic SFN; anti-MX1, anti-DBNL and anti-KRT8 (176). MX1 is an interferon-induced GTP-binding protein, DBNL (drebrin-like protein) is an adapter protein playing a role in endocytosis and synapse formations, and KRT8 (keratin type II cytoskeletal 8) is a contractile apparatus to dystrophin (176). Those antibodies need to be further validated and their pathogenicity is currently not known.

## Peripheral hyperexcitability syndromes

Peripheral hyperexcitability syndromes can concern the nerve or rarely the muscle.

### Peripheral nerve hyperexcitability syndromes

Peripheral nerve hyperexcitability syndromes (PNHS) are a group of rare pathologies characterized by spontaneous and continuous muscle activity, including muscle spasm, stiffness and pain. Primary forms include Isaacs syndrome, Morvan syndrome and cramp fasciculation syndrome. Cramp fasciculation syndrome is the least severe form of the spectrum, and is characterized by episodic cramping, fasciculations, stiffness and muscle pain. Morvan and Isaacs syndromes are characterized by relatively specific neuromyotonic discharges in needle myographies, but also cramps and myokymia. Morvan’s syndrome is also commonly associated with encephalopathy and dysautonomia.

PNHS are usually associated with anti-CASPR2-ab, more rarely LGI1-ab; these antibodies were historically described as targeting the voltage-gated potassium channels (VGKC) complex (177). They can be detected in the serum and the cerebrospinal fluid (CSF), but in case of peripheral nervous system presentation, sensitivity appears to be better in the serum. CASPR2/LGI1 antibodies are described in 0–24% of cramp fasciculation syndromes, 20–30% of Isaacs syndromes and 60–75% of Morvan syndromes (178). However, they are not specific for PNHS, and can be found in limbic encephalitis, movement disorders, epileptic seizures, etc. (179). CASPR2 is a cell-surface adhesion molecule, a critical component of the VGKC complex, present in myelinated axons (180). In mice models, antibodies against CASPR2 were shown to decrease expression of VGKC in dorsal root ganglion neurons and juxtaparanodes, causing pain-related hypersensitivity (180). Another animal model showed that CASPR2 antibodies can also alter CASPR2 protein function in the hippocamp, explaining central nervous system involvement (181). LGI1 is a neuronally secreted molecule, which has been proven to have an essential role in central nervous system neuronal hyperexcitability, through action on VGKC, and in synaptic transmission through AMPA receptors (182). It has been shown that LGI1 is also highly expressed in dorsal root ganglia and spinal cord dorsal horn neurons in mice and human (183). A considerable proportion of patient with Isaac and Morvan syndrome, especially the seropositive case for CASPR2 antibodies, have underlying thymoma (181). These antibodies are included in screening panels for antineural antibodies and are generally detected by CBA, then confirmed by TBA.

### Immune-mediated rippling muscle disease

Rippling muscle disease is a generally benign, myotonic-like myopathy associated with rapid rolling contractions and percussion-induced contractions, caused by a muscle hyperexcitability. One of the main characteristics is that those visible contractions are electrically silent during an EMG recording (184). Genetic forms have been reported, caused by a pathogenic variant in caveolin-3 (*CAV3*) or less frequently cavin-1 (*CAVIN1*) genes (185). In 2022, immune-mediated forms of rippling muscle disease have been described, associated with cavin-4 IgG in 80% of the cases, most of them responding to immunotherapy (186). More recently, a paraneoplastic immune-mediating rippling muscle disease has been described, associated with thymoma (187).

**Figure 1:**
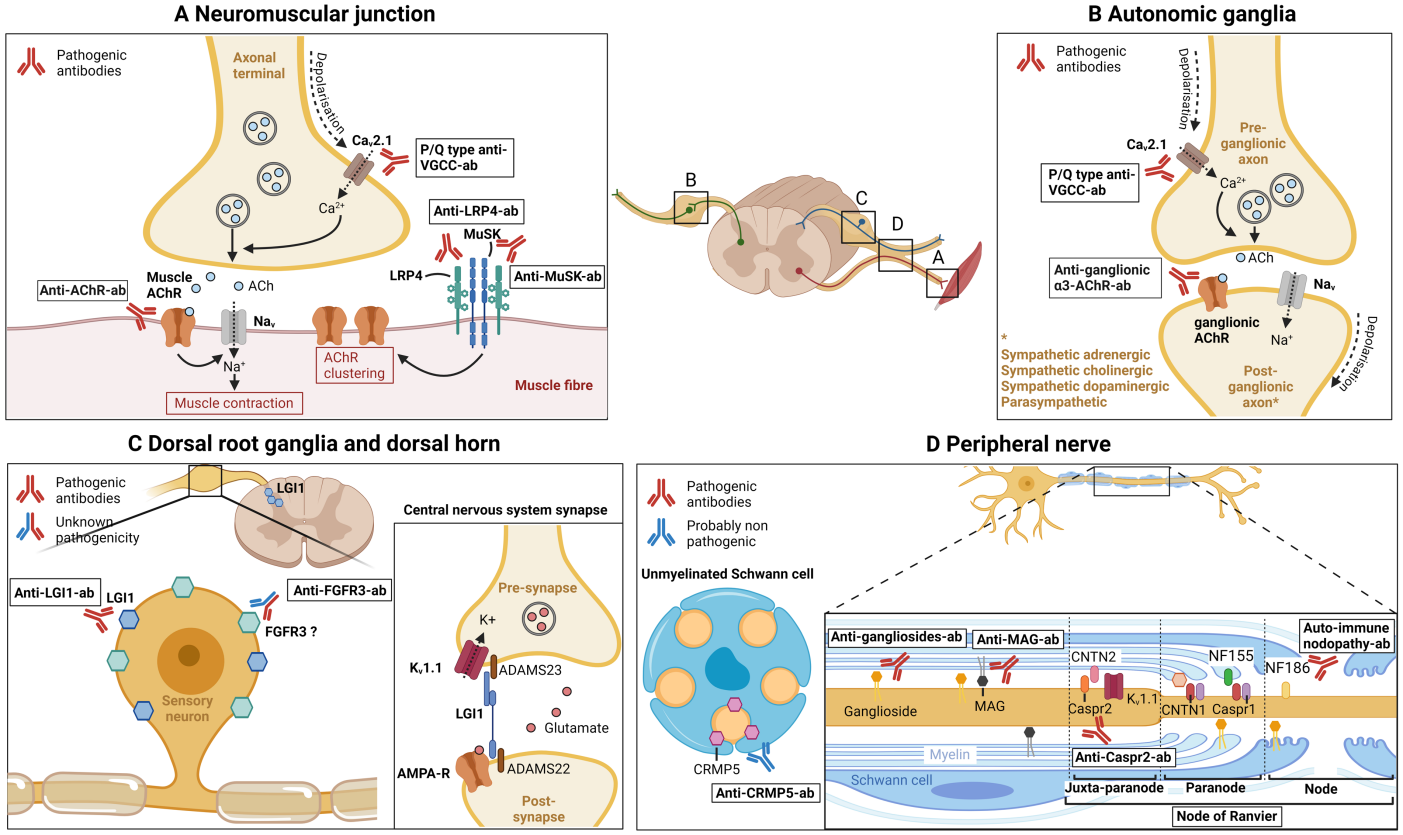
Main antibodies in neuromuscular disorders. (A) Neuromuscular junction. P/Q type anti-VGCC antibodies are directed against the voltage-gated calcium channel (VGCC or Cav2.1) in the axonal terminal, thus blocking calcium influx, acetylcholine vesicle fusion, and neuromuscular transmission. Anti-AChR antibodies target the nicotinic acetylcholine muscle receptor, preventing muscular membrane depolarization and generation of muscle action potential. Anti-LRP4 and anti-MuSK antibodies target the LRP4/MuSK complex, implicated in formation and maintenance of the neuromuscular junction and in acetylcholine receptor clustering. (B) Autonomic ganglia. Anti-ganglionic-α3-AChR antibodies are directed against the ganglionic acetylcholine receptor, preventing post-synaptic depolarization, blocking autonomic neurotransmission. P/Q type anti-VGCC antibodies also blocks the Cav2.1 present in presynaptic axonal terminal. (C) Dorsal root ganglia and dorsal horn. Leucin-rich glioma inactivated 1 (LGI1) was shown to be expressed in dorsal root ganglia and spinal cord dorsal horn, and is the target of anti-LGI1 antibodies. In central nervous system synapses, LGI1 binds to ADAM22/23 at the presynaptic side and modulate Kv1.1 (or voltage gated potassium channel; VGKC) channel; at the post-synaptic side, LGI1 binds to ADAM22 and modulate AMPA receptor. LGI1 therefore modulates central nervous system synaptic transmission. Anti-FGFR3 antibodies are directed against fibroblast growth factor receptor 3, which was shown to be expressed in small and large sensory neurons of the dorsal root ganglia in adult rats. Pathogenicity of anti-FGFR3 antibodies is currently unknown. (D) Peripheral nerve. Anti-gangliosides antibodies target gangliosides, glycoproteins that are expressed on neuronal membranes, Schwann cells and myelin. Anti-MAG antibodies recognizes myelin associated glycoprotein, a glycoprotein localized in periaxonal Schwann cells, which has function in glia-axon interaction. At the nodal and paranodal region, contactin 1, contactin-associated protein 1, neurofascin 155 and neurofascin 186, have different function such as axon-myelin and axon-Schwann cell binding and sodium channel clustering. Antibodies against paranodal/nodal proteins are responsible for auto-immune nodopathies. Contactin-associated protein 2 is expressed in the juxtaparanodal region (and also in dorsal root ganglia), connects to contactin 2 and organizes Kv1.1 (VGKC). Antibodies directed against Caspr2 cause peripheral nerve hyperexcitability. Collapsin response mediator protein-5 has been shown to be expressed in unmyelinated Schwann cells, and is involved in axon-Schwann cell interactions. Antibodies against CRMP5 are probably nonpathogenic, and a marker of paraneoplastic neurological syndrome. ACh, acetylcholine; AChR, Acetylcholine receptor; Cav2.1, voltage-dependent calcium channel 2.1; Caspr1, contactin-associated protein 1; Caspr2, contactin-associated protein 2; CNTN1, contactin 1; CNTN2, contactin 2; CRMP5, Collapsin response mediator protein-5; FGFR3, fibroblast growth factor 3; Kv1.1, voltage-gated potassium channel; LRP4, lipoprotein-related protein 4; MAG, myelin associated glycoprotein; MuSK, muscle kinase; NF155, neurofascin-155; NF186, neurofascin-186; VGCC, voltage gated calcium channel; VGKC, voltage-gated potassium channel.

Characteristics of antibodies associated with neuronopathies, small fiber neuropathies, motor neuron diseases and peripheral hyperexcitability syndromes are summarized in Table 4. The location, characteristics, and mechanism of action of the main antibodies and their antigens involved in neuromuscular junction and peripheral nerve disorders are summarized in Figure 1.

**Table 4:** Characteristics of antibodies associated with neuronopathies, small fiber neuropathies, motor neuron diseases, and peripheral hyperexcitability syndromes.

Disease	Antibody	Detection method	Sensitivity	Specificity	Indication
AAG	Ganglionic AChR type α3	RIA, LIP and immunomodulation assay CBA (not routinely available)	RIA: ~50% of AAG CBA: probably as sensitive as RIA	RIA: >1.0 nmoL/L: high specificity 0.2–1.0 nmoL/L: moderate specificity <0.2 nmoL/L: nonspecific CBA: probably very specific	Subacute and severe autonomic failure
SNN	FGFR3	ELISA	~15% of sensory neuropathies	Probably specific to sensory neuropathies (and not neuronopathies)	Subacute SNN with negative workup and suspicion of dysimmune etiology
	AGO1		~13% of SNN	Probably low (false+ in systemic and CNS auto-immune diseases)	
	ANNA-1	Immunoblot confirmed by TBA	82% of paraneoplastic SNN More sensitive in the serum than CSF	99% of paraneoplastic SNN More specific if also+ in the CSF	First line workup of SNN, especially if subacute and severe
	CRMP5		Unknown More sensitive in the serum than CSF	Good (not specific to SNN) More specific if also+ in the CSF	
MND	IgLON5	Immunoblot confirmed by TBA	Unknown	Unknown	Bulbar-onset ALS with sleep disorders, vocal cord paresis or chorea
SFN	FGFR3	ELISA	15% of patients with cryptogenic SFN	Probably specific to sensory neuropathies (not neuronopathies)	Unknown (suspected auto immune SFN?)
	TS-HDS		37% of patients with cryptogenic SFN	Not specific to SFN (found in healthy controls)	
	Plexin D1		13% of patients with probable SFN	Probably specific to SFN	
PNHS	CASPR2/LGI1	CBA confirmed by TBA	0–24% of CFS 20–30% of Isaacs syndromes 60–75% of Morvan syndromes	Good (not specific to PNHS)	Every suspicion of PNHS
RMD	Cavin-4	CBA (not routinely available)	80% of immune-mediated RMD, according to one study	Unknown	Every RMD?

AAG, auto-immune autonomic ganglionopathy; AChR, acetylcholine receptor; AGO1, argonaute-1; ANNA-1, antineuronal nuclear antibody type 1; CASPR2, contactin associated protein 2; CBA, cell-based assay; CFS, cramp-fasciculation syndromes; CNS, central nervous system; CRMP5, collapsing response mediator protein-5; ELISA, enzyme-linked immunosorbent assay; FGFR3, fibroblast growth factor 3; IgLON5, Ig-like cell adhesion molecule 5; LGI1, leucine-rich glioma inactivated 1; LIP, Luciferase immunoprecipitation assay; PAF, pure autonomic failure; POTS, postural orthostatic tachycardia syndrome; PNHS, peripheral nerve hyperexcitability syndromes; RMD, rippling muscle disease; RIA, radio-immunoprecipitation assay; SFN, small-fiber neuropathy; SNN, sensory neuronopathy; TBA, tissue-based assay; TS-HDS, Trisulfated heparan disaccharide.

## Idiopathic inflammatory myopathies

Idiopathic inflammatory myopathies (IIM), also known as myositis, are a heterogeneous group of autoimmune diseases affecting multiples organs, including muscles, skin, lungs and joints (188, 189). Muscle involvement is typically characterized by subacute proximal weakness, sometimes associated with muscle pain. A major advance in the field of IIM is the discovery of myositis-specific autoantibodies, present in up to 60% of patients with IIM (190). Those antibodies are specific for myositis and are strongly associated with distinct clinical phenotypes. In addition, patients with IIM may be positive for autoantibodies that are present in other autoimmune disorders (systemic lupus erythematosus, systemic sclerosis etc.); they are often named myositis-associated auto-antibodies (189). Based on a combination of myositis-specific autoantibodies, clinical presentation, muscle MRI pattern and muscle biopsy features, patients can be classified in five distinct subtypes; dermatomyositis, antisynthetase syndrome, overlap myositis, immune-mediated necrotizing myopathy (IMNM) and inclusion-body myositis (IBM) (191, 192). The old and imprecise term “polymyositis” does not correspond to a precise entity, since more than 90% of polymyositis turn out to be IBM or IMNM upon follow-up.

### Dermatomyositis

Dermatomyositis (DM) is defined by the presence of characteristic cutaneous manifestations (heliotrope rash, V sign rash, Gottron signs and papules etc.) and myositis, which can be inconstantly associated (189). Most patients with DM (~70%) have a myositis-specific antibody: anti-Mi-2, anti- MDA5, anti-TIF1, anti-NXP2 or anti-SAE (189). Each autoantibody is associated with a distinct clinical phenotype, leading to a subclassification of DM according to those antibodies (191). These associations have important clinical impact: patients with anti-TIF1 and -NXP2-ab have a higher risk of oncological disease, whereas anti-Mi-2 present with milder myositis. Anti-MDA-5 and -SAE are usually amyopathic but may have a dramatic course with severe interstitial lung disease or severe dysphagia, respectively.

### Anti-synthetase syndrome

Anti-synthetase syndrome (ASyS) is a relatively homogeneous multisystem disease, usually classified as IIM although myositis is not always present. The most common manifestation is interstitial lung disease, which also determines the functional outcome and prognosis. ASyS is characterized by auto-antibodies against one of many amino-acyl transfer RNA (tRNA). Eight auto-antibodies have been identified: anti-Jo1, anti-Ha/YRS, anti-Zo, anti-EJ, anti-PL-7, anti-OJ, anti-KS and anti-PL-12 (190, 191).

### Immune-mediated necrotizing myopathy

Immune-mediated necrotizing myopathy (IMNM) are characterized by a proximal symmetric and axial myopathy, usually extremely high muscle enzyme levels, and muscle biopsy showing necrosis or regeneration with minimal lymphocytic infiltrate (193). Patients rarely have prominent systemic manifestations. Patients with IMNM usually (60–70%) have auto-antibodies recognizing either 3-hydroxy-3-methylglutaryl-coenzyme A reductase (HMGCR) or the signal recognition particle (SRP). According to those autoantibodies, IMNM can be classified as anti-HGMCR, anti-SRP and auto-antibody-negative IMNM, each subtype being a specific entity. Patients with anti-SRP usually have a more severe myopathy, extra-muscular manifestations (including cardiac involvement) and higher chance of dysphagia (194). The risk of developing anti-HMGCR IMNM is higher following statin exposure, and patients usually have a severe myopathy, without extra-muscular manifestations; the risk of developing cancer is higher in anti-HMGCR and seronegative IMNM (194).

### Overlap myositis

Myositis can occur with other connective tissue disease such as systemic lupus erythematosus, systemic sclerosis, Sjögren syndrome or rheumatoid arthritis. Autoantibodies detected in overlap myositis include anti-U1RNP, anti-Ku, anti-PM-Scl, anti-RuvBL1, anti-RuvBL2, anti-Ro/SSA and anti-La/SSB (189, 195).

### Inclusion-body myositis

Inclusion-body myositis (IBM) is characterized by asymmetrical weakness of both proximal and distal muscles that predominates on the quadriceps and long finger flexors, with a very slowly progressive course, and occurs mainly in individuals >50 years of age. As of the ENMC diagnostic criteria, typical clinical presentation and histological feature (endomysial infiltrate surrounding non necrotic fibers) are sufficient to make a diagnosis. In case of incomplete or atypical clinical picture, supportive criteria are required. These criteria include other histopathological features, imaging studies, and the positivity of autoantibodies recognizing cytosolic 5′-nucleotidase 1A (cN1a) antibodies (196). Anti-cN1a-ab have been described in 2013 (197) and may be detected in 33–80% of patients with IBM (198, 199). In initial studies, the specificity of those antibodies has been reported in the range of 92–100% (199). However, more recent studies showed that anti-cN1a-ab can also been found in patients with IIM (PM, DM, IMNM), SLE and Sjögren syndrome (198, 200). Various detection method can be used (ELISA, WB, CBA, immunoprecipitation), and the best method is currently unknown (199, 200).

### Detection of IIM antibodies

Detection of myositis-specific antibodies and myositis-associated antibodies can be made with various detection methods. As many of those antibodies target cytoplasmic antigens, a weak or negative nuclear staining is achieved with indirect immunofluorescence using Hep2 cells. Indirect immunofluorescence has therefore limited utility as screening test. Immunoprecipitation is probably the gold-standard for most myositis autoantibodies detection, but is time-consuming, expensive and availability is limited to specialized centers (201). ELISA is a reliable method, but not all myositis-specific and myositis-associated antibodies are amenable to this testing (202). In the recent years, commercial line immunoblot assay and immunodot assay have been developed and have improved availability and diagnostic accuracy of myositis subtypes (203). Two of those commercial immune assays have been assessed and compared to immunoprecipitation in a recent study. Overall, those assays performed relatively well with sensitivity and specificity varying according to antibodies, with two notable exceptions: a poor detection rate of anti-TIF1γ (40% of false negative with line blot and 76% of false negative with dot blot) and a poor detection rate of rare anti-synthetase autoantibodies (200). In addition, a high false-positive rate was observed, in 13.7% of samples analyzed by line blot and 12.1% analyzed by dot blot, usually at low titer (201). Results of those commercial immunoassays must therefore be interpreted within the clinical context, and in selected cases, immunoprecipitation should be considered.

Myositis-specific and associated antibodies need to be tested in every suspicion of inflammatory myopathy. As detection of such antibody is part of the classification process of IMM, a positive antibody will help guide investigations (e.g., paraneoplastic workup) and guide treatment. It is important to note that the diagnostic value of myositis-specific and myositis-associated antibodies depends on pre-test probability, determined mainly by the clinical pictures and complementary exams like electromyography (204).

Characteristics of antibodies associated with IIM are summarized in Table 5.

**Table 5:** Characteristics of antibodies associated with autoimmune myopathies.

Disease	Antibodies	Recommended detection method	Sensitivity	Specificity	Indication	Comment
DM	Mi-2, MDA5, TIF1, NXP2 and SAE	IP (not routinely available) Immunoblot or immunodot	~70% of DM Sensitivity of immunoblot/immunodot vary according to antibodies (i.e., low sensitivity of anti-TIF1γ)	~80–90% (frequent false + with immunoblot/immunodot, especially at low titers)	Every suspicion of idiopathic inflammatory myopathy	TIF1/NXP2: high association with tumor MDA5/SAE: usually amyopathic
ASyS	Jo1, Ha/YRS, Zo, EJ, PL-7, OJ, KS and PL-12		Good sensitivity?			
IMNM	HMGCR and SRP		60–70% of IMNM			SRP: severe myopathy, extramuscular involvement and frequent dysphagia HMGCR: severe myopathy, no extramuscular involvement, high association with tumor
OM	U1RNP, Ku, PM-Scl, RuvBL1, RuvBL2, Ro/SSA and La/SSB		Good sensitivity?			
IBM	cN1a	ELISA, WB, CBA or IP	33–80% of IBM	~90% (false + in other IIM, SLE or Sjögren)	In case of incomplete or atypical cases	

ASyS, antisynthetase antibody syndrome; CBA, cell-based assay; DM, dermatomyositis; ELISA, enzyme-linked immunosorbent assay; IBM, inclusion-body myositis; IIM, idiopathic immune myopathies; IMNM, immune mediated necrotizing myopathy; IP, immunoprecipitation; OM, overlap myositis; SLE, systemic lupus erythematosus; WB, western blot.

## Conclusion

Autoantibody testing is extremely useful in the management of a great number of patients with inflammatory neuromuscular disorders (205). In addition to confirming a diagnosis, the presence of such autoantibody can guide the paraneoplastic workup (e.g., the screening for thymoma in anti-AChR+ MG) and treatment (e.g., anti-C5 therapy in anti-AChR+ MG, anti-CD20 therapy in anti-MuSK+ MG and autoimmune nodopathy or IVIg therapy in anti-FGFR3+ sensory neuronopathy). Moreover, the recent discovery of new autoantibodies (such as anti-nodal and paranodal antibodies in auto-immune nodopathies, anti-FGFR3 and AGO1 in sensory neuronopathies or anti-cN1a in inclusion-body myositis) has led to a better understanding of the pathophysiology of immune-mediated neuromuscular diseases.

Despite their high clinical usefulness, clinicians should be aware of limitations related to such autoantibodies screening. First, their specificity is not always optimal. For example, anti-LRP4 antibodies are not specific to MG and may be found in patients with ALS. Anti-gangliosides antibodies are non-specific at low titers, and only some antibodies are associated with a specific clinical phenotype. False positives are frequent when testing for muscle-specific and muscle-associated autoantibodies. Finally, when testing for autoantibodies, clinicians should be aware of the used detection method. Indeed, sensitivity and specificity of detection techniques can vary widely, and the use of the reference method should be encouraged. However, some of these techniques (such as immunoprecipitation for the detection of muscle autoantibodies) are not widely available in clinical practice.
